# Biochemical impact and therapeutic efficacy of laparoscopic surgery in early-stage ovarian cancer: Modulation of vascular endothelial growth factor and tumor markers

**DOI:** 10.5937/jomb0-56011

**Published:** 2025-06-13

**Authors:** Peng Han, Yafei Liu, Jin Han

**Affiliations:** 1 The Second Affiliated Hospital of Xingtai Medical College, Xingtai, China; 2 The First Affiliated Hospital of Xingtai Medical College, Xingtai, China

**Keywords:** laparoscopic surgery, open surgery, early ovarian cancer, vascular endothelial growth factor, tumor markers, angiogenesis, biochemical markers, enzyme-linked immunosorbent assay, laparoskopska hirurgija, otvorena hirurgija, rani rak jajnika, faktor rasta vaskularnog endotela, tumorski markeri, angiogeneza, biohemijski markeri, enzimski imunosorbentni test

## Abstract

**Background:**

The objective of this study was to investigate the biochemical impact of laparoscopic surgery on angiogenesis, focusing on vascular endothelial growth factor (VEGF), and the modulation of key tumor markers in patients with early-stage ovarian cancer.

**Methods:**

A total of 76 patients diagnosed with early ovarian cancer were enrolled and divided into two groups based on the surgical approach: the control group (n=38) underwent open surgery, and the observation group (n=38) underwent laparoscopic surgery. Surgical parameters, VEGF levels, tumor markers [matrix metalloproteinase 9 (MMP9), stromal cell-derived factor-1a (SDF-1a), and carcinoembryonic antigen (CEA)], survival rates, and incidence of complications were compared between the two groups.

**Results:**

The duration of surgery was longer in the observation group than in the control group. However, the hospitalization time, recovery of bowel function, and length of surgical incision were significantly shorter in the observation group. Intraoperative blood loss was also significantly lower in the observation group, with all differences being statistically significant (P<0.01). Seven days post-surgery, VEGF, MMP9, SDF-1a, and CEA levels in both groups decreased compared to their preoperative levels. These levels were significantly lower in the observation group than in the control group (P<0.05). The 24-month survival rate was higher in the observation group (P<0.05). There was no statistically significant difference in the total incidence of complications between the two groups (P>0.05).

**Conclusions:**

Laparoscopic surgery for early ovarian cancer results in minimal trauma, reduces VEGF and tumor marker levels, and improves the 24-month survival rate without increasing the incidence of complications.

## Abstract (sr)

**Uvod:**

Cilj ove studije je bio da se ispita biohemijski uticaj laparoskopske hirurgije na angiogenezu, sa fokusom na faktor rasta vaskularnog endotela (VEGF) i modulaciju ključnih tumorskih markera kod pacijenata sa ranim stadijumom raka jajnika.

**Metode:**

Ukupno 76 pacijenata sa dijagnostikovanim ranim karcinomom jajnika je upisano i podeljeno u dve grupe na osnovu hirurškog pristupa: kontrolna grupa (n=38) je podvrgnuta otvorenom operaciji, a posmatračka grupa (n=38) je podvrgnuta laparoskopskoj operaciji. Hirurški parametri, nivoi VEGF, tumorski markeri [matriks metaloproteinaza 9 (MMP9), faktor-1a (SDF-1a) izveden iz stromalnih ćelija (SDF-1a) i karcinoembrionalni antigen (CEA)], stope preživljavanja i učestalost komplikacija upoređivani su između dve grupe.

**Rezultati:**

Trajanje operacije bilo je duže u posmatranoj grupi nego u kontrolnoj grupi. Međutim, vreme hospitalizacije, oporavak funkcije creva i dužina hirurškog reza bili su značajno kraći u posmatranoj grupi. Intraoperativni gubitak krvi je takođe bio značajno manji u posmatranoj grupi, pri čemu su sve razlike statistički značajne (P<0,01). Sedam dana nakon operacije, nivoi VEGF, MMP9, SDF-1a i CEA u obe grupe su se smanjili u poređenju sa njihovim preoperativnim nivoima. Ovi nivoi su bili značajno niži u posmatranoj grupi nego u kontrolnoj grupi (P<0,05). Stopa preživljavanja od 24 meseca bila je veća u posmatranoj grupi (P<0,05). Nije bilo statistički značajne razlike u ukupnoj incidenci komplikacija između dve grupe (P>0,05).

**Zaključak:**

Laparoskopska hirurgija ranog karcinoma jajnika dovodi do minimalne traume, smanjuje nivoe VEGF-a i tumorski markera i poboljšava 24-mesečnu stopu preživljavanja bez povećanja incidence komplikacija.

## Introduction

Ovarian cancer is one of the most lethal malignancies affecting women, with high mortality ratesattributed to its asymptomatic nature in early stages and frequent late-stage diagnoses [1] [2] [3]. The development of ovarian cancer is influenced by various factors, including genetic predisposition, hormonal influences, reproductive history, and environmental exposure [4] [5] [6]. Genetic mutations, such as those in BRCA1 and BRCA2 genes, significantly increase risk, while protective factors include the use of oral contraceptives and pregnancies [7] [8] [9]. Despite these known risk factors, early detection remains a major challenge, as many patients present with vague symptoms such as abdominal discomfort or bloating [10]. Consequently, ovarian cancer continues to rank among the leading causes of cancer-related deaths in women globally [11].

For patients diagnosed with early-stage ovarian cancer, surgical intervention is the primary treatment modality. Open surgery has been the traditional approach, providing comprehensive tumor resection and prolonged survival. However, it is associated with significant trauma, prolonged recovery, and a higher risk of complications, such as infections and delayed wound healing [12]. Laparoscopic surgery, a minimally invasive alternative, has gained attention due to its reduced surgical trauma, faster recovery, and lower complication rates [13] [14] [15]. However, the biochemical impacts of laparoscopic surgery, particularly its effects on vascular endothelial growth factor (VEGF) and tumor markers, require further investigation.

VEGF plays a pivotal role in tumor angiogenesis, driving endothelial cell proliferation and migration, which accelerate tumor growth and metastasis [16] [17]. Similarly, tumor markers such as matrix metalloproteinase-9 (MMP9), stromal cell-derived factor-1α (SDF-1α), and carcinoembryonic antigen (CEA) are essential indicators of tumor progression and response to treatment [18] [19] [20]. Elevated levels of these markers are closely associated with poor clinical outcomes in ovarian cancer patients. While previous studies have highlighted the clinical benefits of laparoscopic surgery, its influence on VEGF and tumor marker modulation remains insufficiently explored.

Given these considerations, this study aims to evaluate the biochemical and clinical outcomes of laparoscopic versus open surgery in patients with early-stage ovarian cancer. By analyzing surgicalparameters, VEGF levels, tumor markers, and survival outcomes, the study seeks to provide comprehensive insights into the potential advantages of laparoscopic surgery, thereby guiding future treatment strategies for early-stage ovarian cancer.

## Materials and methods

### Patient selection

This study included patients with early-stage ovarian cancer who were admitted to our hospital between January 2018 and December 2022. The inclusion criteria for participation required patients to meet the diagnostic criteria for ovarian cancer, with the diagnosis confirmed through pathological examination. Additionally, only those patients who were eligible for either open or laparoscopic surgery and had no contraindications for these procedures were included. The clinical stage of the cancer for all patients was limited to stages I or II, according to the International Federation of Gynecology and Obstetrics (FIGO) guidelines.

Patients were excluded from the study if they had received chemotherapy, radiotherapy, orimmuno therapy before surgery. Other exclusion criteria included abnormal coagulation function, hepatic or renal impairment, the presence of malignancies in other organs, or a history of previous abdominal or pelvic surgery. A total of 76 patients met the eligibility criteria and were enrolled in the study. These patients were divided into two groups based on the surgical method employed. The control group, comprising 38 patients, underwent open surgery, while the observation group, consisting of 38 patients, received laparoscopic surgery. The two groups were comparable in terms of demographic and clinical characteristics, including age, body mass index (BMI), tumor diameter, and FIGO stage, with no statistically significant differences observed between them (P > 0.05). This study was approved by the ethics committee of our hospital. Signed written informed consents were obtained from the patients and/or guardians.

### Surgical procedures

### Open surgery

Patients in the control group underwent open surgery following standard preoperative assessments. These assessments included bowel preparation and correction of any metabolic imbalances. After the patients were placed under general anesthesia with endotracheal intubation, a midline abdominal incision approximately 15 cm in length was made. Tumor resection was performed under direct visualization to ensure complete removal. Adhesions and the omentum were separated and removed as part of the surgical procedure. Lymph node dissection was performed in the abdominal and pelvic regions to identify any metastatic involvement. Additionally, the uterus, ovaries, and appendix were excised to achieve comprehensive tumor resection. Any visible metastatic lesions were resected as well to ensure no remaining cancerous tissues.

### Laparoscopic surgery

Patients in the observation group underwent laparoscopic surgery, which included total hysterectomy, bilateral salpingo-oophorectomy, omentectomy, appendectomy, and pelvic and para-aortic lymph node dissection. After general anesthesia was administered, a 10-mm transverse incision was made above the umbilicus to insert the first trocar and establish pneumoperitoneum at a pressure of 13 mmHg. The abdominal and pelvic organs were carefully inspected using the laparoscope, taking advantage of the magnified view to detect any abnormalities. Additional incisions were made to insert trocars and surgical instruments required for the procedure. Fluid samples were aspirated from the abdominal cavity for cytological examination to rule out the presence of cancer cells. Any suspicious lesions identified during the inspection were excised and placed in protective bags for extraction through the incisions. Routine postoperative care, including anti-inflammatory treatment and nutritional support, was provided to all patients in both groups to promote recovery.

### Outcome measures

The primary outcome measures included surgical parameters, levels of vascular endothelial growth factor (VEGF) and tumor markers, and survival rates with associated complications. The surgical parameters assessed were the duration of the surgery, the length of hospital stay, the number of lymph nodes dissected, the time required for bowel function recovery, the amount of intraoperative blood loss, and the length of the surgical incision.

The levels of VEGF, MMP9, SDF-1α, and CEA were quantified using enzyme-linked immunosorbent assays (ELISA) following the manufacturer’s protocols. The assays were performed in duplicate toensure accuracy, with appropriate controls included in each batch. The sensitivity and specificity of the assay kits were verified against standard curves, and interassay variability was controlled within 5%.

The survival outcomes of the patients were monitored over a two-year follow-up period. Survival rates were assessed at 8, 16, and 24 months after surgery to determine the effectiveness of each surgical technique.

### Postoperative complications monitoring and categorization

Postoperative complications were monitored and recorded for all patients during their hospital stay and at follow-up visits at 1, 3, 6, 12 and 24 months post-surgery. Monitoring included routine clinical examinations, patient-reported symptoms, and diagnostic evaluations such as blood tests, imaging studies (e.g., ultrasound or CT scans), and microbiological cultures for suspected infections. Complications were categorized using the Clavien-Dindo classification system to ensure consistency and transparency. This system grades complications based on their severity, ranging from minor complications requiring no intervention (Grade I) to life-threatening events requiring major surgical or medical intervention (Grade IV) and death (Grade V). Specific complications monitored included: Wound infections: Diagnosed based on the presence of erythema, purulent discharge, and positive wound cultures; Urinary retention: Defined as the inability to void spontaneously, confirmed by post-void residual volumes exceeding 100 mL on ultrasound; Deep vein thrombosis (DVT): Diagnosed using Doppler ultrasonography in patients presenting with unilateral leg swelling, pain, or other suggestive symptoms. All complications were independently reviewed by two senior clinicians to ensure accuracy in classification and reporting. The total incidence of complications was compared between the laparoscopic and open surgery groups to evaluate the safety profiles of each approach.

### Statistical analysis

All statistical analyses were performed using SPSS 24.0 software. Categorical variables were presented as counts and percentages, and comparisons between the two groups were conducted using the chi-square test. Continuous variables were expressed as mean ± standard deviation (x̄±s), and comparisons between groups were analyzed using the t-test. A P-value of <0.05 was considered to indicate a statistically significant difference between the two groups. These statistical methods ensured that the data were analyzed rigorously and the results were interpreted accurately.

## Results

### Comparison of surgical parameters

There was no significant difference in the number of lymph nodes dissected between the two groups (P>0.05). However, the operation time was significantly longer in the observation group compared to the control group. The observation group also exhibited significantly shorter hospital stays, faster bowel recovery, smaller incisions, and lower intraoperative blood loss than the control group (P<0.01) (Table 1).

**Table 1:** Comparison of Surgical Parameters between Laparoscopic and Open Surgery Groups.

Parameter	Laparoscopic Surgery Group<br>(n=38)	Open Surgery Group<br>(n=38)	t-value	P-value
Operation time (min)	246.14 ± 34.85	203.21 ± 26.43	6.724	<0.01
Hospital stay (days)	14.78 ± 2.10	18.73 ± 3.48	6.523	<0.01
Lymph nodes dissected<br>(number)	32.87 ± 4.51	33.02 ± 4.37	0.137	0.892
Bowel function recovery<br>(hours)	5.27 ± 0.89	9.48 ± 0.96	22.309	<0.01
Intraoperative blood loss<br>(mL)	118.62 ± 11.12	214.57 ± 16.09	33.971	<0.01
Incision length (cm)	6.95 ± 0.67	16.47 ± 2.03	27.872	<0.01

### Biochemical changes in VEGF and tumor markers

Before surgery, there were no significant differences in VEGF, MMP9, SDF-1α, and CEA levels between the two groups (P>0.05). Seven days postsurgery, these levels decreased in both groups. However, the observation group had significantly lower VEGF, MMP9, SDF-1α, and CEA levels compared to the control group (P<0.05) (Table 2).

**Table 2:** Comparison of VEGF and Tumor Marker Levels Pre- and Post-Surgery.

Parameter	Time	Laparoscopic Surgery<br>Group (n=38)	Open Surgery Group<br>(n=38)	t-value	P-value
VEGF (pg/mL)	Pre-surgery	174.18 ± 12.25	172.37 ± 13.42	0.283	0.779
	7 days post-surgery	93.05 ± 9.87	116.18 ± 12.21	9.694	<0.05
MMP9 (ng/mL)	Pre-surgery	342.12 ± 15.21	340.34 ± 15.53	0.513	0.608
	7 days post-surgery	190.07 ± 13.64	226.42 ± 15.71	10.143	<0.05
SDF-1α (ng/mL)	Pre-surgery	65.21 ± 5.34	64.89 ± 5.23	0.259	0.796
	7 days post-surgery	31.82 ± 3.91	47.67 ± 4.19	16.175	<0.05
CEA (ng/mL)	Pre-surgery	3.15 ± 0.59	3.22 ± 0.63	0.472	0.638
	7 days post-surgery	1.82 ± 0.43	2.43 ± 0.48	6.725	<0.05

### Survival rates and complications

There were no significant differences in survival rates at 8 and 16 months between the two groups. However, the 24-month survival rate was significantly higher in the observation group (P=0.025). The overall incidence of complications was similar between the groups, with 4.55% in the observation group and 9.30% in the control group (Table 3).

**Table 3:** Comparison of Survival Rates and Complication Incidence.

Parameter	Laparoscopic Surgery Group<br>(n=38)	Open Surgery Group<br>(n=38)	χ^2^-value	P-value
8-month survival rate (%)	92.11	89.47	1.037	0.308
16-month survival rate (%)	84.21	80.26	0.597	0.439
24-month survival rate (%)	78.95	63.16	5.054	0.025
Complication rate (%)	5.26	10.53	0.764	0.382

## Discussion

This study demonstrated that laparoscopic surgery for early-stage ovarian cancer is associated with significant biochemical and clinical benefits, including reduced vascular endothelial growth factor (VEGF) and tumor marker levels, shorter recovery times, and improved 24-month survival rates. These findings align with previous research indicating that laparoscopic surgery minimizes surgical trauma and facilitates faster recovery compared to open surgery [13] [14] [15]. Importantly, our study extends this understanding by highlighting the modulation of key biochemical markers, including VEGF, matrix metalloproteinase-9 (MMP9), stromal cell-derived factor-1α (SDF-1α), and carcinoembryonic antigen (CEA), which are closely linked to tumor progression and angiogenesis.

The reduction in VEGF levels observed in the laparoscopic group is consistent with studies demonstrating the role of minimally invasive techniques in suppressing angiogenesis [16] [17]. VEGF is a critical regulator of tumor angiogenesis, promoting endothelial cell proliferation and migration. By reducing VEGF levels, laparoscopic surgery may mitigate angiogenesis, thereby slowing tumor growth and metastasis. Similarly, the observed decreases in MMP9 and SDF-1α levels suggest a suppression of extracellular matrix degradation and chemotactic signaling, which are key processes in tumor invasion and metastasis [18] [19].[20] These results underscore the potential of laparoscopic surgery to achieve superior biochemical outcomes in patients with early-stage ovarian cancer.

Our findings also align with previous studies reporting faster recovery times, reduced intraoperative blood loss, and shorter hospital stays with laparoscopic surgery [13] [14] [15]. However, the longer operation time observed in the laparoscopic group highlights a procedural challenge that may be addressed with advanced training and technology.

While our study provides valuable insights, it has some limitations. The relatively short follow-up period (24 months) may not fully capture long-term survival outcomes or the recurrence rates associated with different surgical approaches. Additionally, the exclusion of patients who had received preoperative chemotherapy, radiotherapy, or immunotherapy limits the generalizability of the findings to broader patient populations. Future studies should include larger, more diverse cohorts and explore the impact of laparoscopic surgery on advanced-stage ovarian cancer and other biomarkers.

From a clinical perspective, our findings support the broader adoption of laparoscopic surgery for early-stage ovarian cancer. The significant reductions in VEGF and tumor marker levels, coupled with improved short-term outcomes, suggest that laparoscopic surgery not only minimizes surgical trauma but also offers biochemical benefits that may enhance long-term survival. Further research is needed to validate these findings and explore the potential of laparoscopic surgery in combination with adjuvant therapies to optimize patient outcomes.

## Conclusions

In conclusion, laparoscopic surgery demonstrates clear advantages over open surgery in terms of biochemical and clinical outcomes for early-stage ovarian cancer. By reducing angiogenesis-related markers and promoting faster recovery, laparoscopic surgery represents a valuable treatment option that warrants further exploration in clinical practice.

## Dodatak

### Conflict of interest statement

All the authors declare that they have no conflict of interest in this work.
